# Obituary

**Published:** 1895-03

**Authors:** 


### Obituary.

Dr. JAMES NICHOLS, of Bradford, Pa., died in that city Saturday morning, February 16, 1895, in the seventieth year of his age. Dr. Nichols was one of the most prominent physicians of his region. He was accomplished in his profession, cultivated in his manners and possessed of a kindly disposition, all of which went to make him a popular physician, a distinguished citizen and a genial companion. He was an alumnus of Buffalo University medical college, and after graduation settled in Limestone, N. Y. In March, 1852, he married Miss Mary J. Wade, of Farmersville, who with two sons and a daughter survive him. One son, Dr. H. J. Nichols, was associated with his father in practice at Bradford. The funeral was held under the auspices of Trinity Commandery Knights Templar, and the interment was at Oak Hill cemetery.

